# Rethinking Cholecystitis: A Case Where Hiccups Told the Story

**DOI:** 10.7759/cureus.82936

**Published:** 2025-04-24

**Authors:** Archit Garg, Abhishek Chouthai, Marcella Pimpinelli, Alana Barofsky, Arkady Broder

**Affiliations:** 1 Internal Medicine, Saint Peter's University Hospital/Rutgers Robert Wood Johnson Medical School, New Brunswick, USA; 2 Gastroenterology, Saint Peter’s University Hospital/Rutgers Robert Wood Johnson Medical School, New Brunswick, USA; 3 Internal Medicine, Saint Peter’s University Hospital/Rutgers Robert Wood Johnson Medical School, New Brunswick, USA; 4 Division of Gastroenterology and Hepatology, Saint Peter’s University Hospital/Rutgers Robert Wood Johnson Medical School, New Brunswick, USA

**Keywords:** acute calculous cholecystitis, acute hiccups, atypical presentation, diabetes mellitus type 2, elderly population, gallstone cholecystitis, intractable hiccups

## Abstract

Acute cholecystitis due to inflammation of the gallbladder typically presents with right upper quadrant abdominal pain, nausea, vomiting, and fever. However, elderly patients with comorbidities can present with atypical symptoms like generalized malaise or confusion, making diagnosis challenging. Here, we present a unique case of acute cholecystitis presenting solely with persistent hiccups. The diagnosis was confirmed with imaging, and it was managed conservatively with a resolution of hiccups. This highlights the importance of keeping broad differentials and using prudent imaging, especially in the elderly, to ensure timely diagnosis and treatment.

## Introduction

Acute cholecystitis is characterized by acute inflammation of the gallbladder, resulting from obstruction of the cystic duct, which subsequently causes chemical or bacterial inflammation of the gallbladder [1]. In 95% of the cases, cystic duct obstruction is caused by gallstones [1]. Acute cholecystitis typically presents as right upper quadrant (RUQ) abdominal pain, nausea, vomiting, anorexia, or fever [2]. The examination may reveal tenderness in the RUQ of the abdomen, voluntary/involuntary guarding on abdomen palpation, tachycardia, and hyperpyrexia [3]. Laboratory testing may show leukocytosis and elevated liver enzymes [4]. Imaging can confirm the diagnosis by the presence of gallbladder inflammation with thickening of the gallbladder wall [4].

However, acute cholecystitis can have a variable presentation and pose a challenge in diagnosis. This is particularly true for elderly patients aged ≥65 years, in whom classical symptoms may be absent [5,6]. Dhir et al. [6] reported two cases of gangrenous cholecystitis and emphasized that elderly subjects can be asymptomatic or have atypical manifestations and are more susceptible to complications from delayed presentation to the emergency [6]. A study conducted by Trowbridge et al. [7] in 2003 showed that there was no single clinical finding or laboratory test that could include or exclude cholecystitis. Studies have shown that acute cholecystitis can present without abdominal pain, with patients' presentations ranging from isolated nausea, fevers, chest pain with ST elevations on electrocardiogram (EKG), or hypertension [5-9]. In such cases, diagnosis can be challenging and delayed.

Hiccups, as a presenting symptom of cholecystitis, are extremely rare. Safe et al. [5] reported a case of intractable hiccups, which on further evaluation showed that the patient had gangrenous cholecystitis. Ongoing inflammation causing diaphragmatic irritation is believed to be the primary factor contributing to the hiccups [5]. Here, we present an extremely uncommon case of acute cholecystitis manifesting solely as persistent hiccups. This case emphasizes the diagnostic challenges posed by such an unusual presentation and underscores the importance of considering unconventional manifestations of disease rather than relying solely on classical symptoms.

## Case presentation

A 74-year-old gentleman with a past medical history of diabetes mellitus type 2, hypertension, and hyperlipidemia, presented to our hospital with the chief complaint of hiccups for three days. The hiccups were sudden and persisted continuously, which prompted him to come to the hospital. He did not endorse any abdominal pain, nausea, vomiting, fevers, chills, jaundice, or cough. On examination, the patient was hemodynamically stable (BP 105/47, HR 87, RR 12, SpO2 99%, Temp 98F). The physical exam was unremarkable without abdominal tenderness, guarding, or rigidity. Murphy's sign was negative. 

The laboratory results showed a normal WBC count (10800/cu.mm), elevated liver enzymes (total bilirubin 5.6 mg/dL, direct bilirubin 1.3 mg/dL, AST 49 IU/L, ALT 102 IU/L, and ALP 284 IU/L), and elevated inflammatory markers (ESR 90 mm/h and CRP 212 mg/L). The laboratory results on admission are shown in Table 1.

**Table 1:** Laboratory test results on admission WBC: White blood cells, BUN: Blood urea nitrogen, ALP: Alkaline phosphatase, AST: Aspartate aminotransferase, ALT: Alanine aminotransferase

Laboratory Test	Value	Reference Range
Hemoglobin	14.7	13 to 17 g/dL
WBC	10.8	4 to 11 x 10^3 /cumm
Platelets	220	1500 to 400 x 10^3 /cumm
BUN	38	9 to 28 mg/dL
Creatinine	2.13	0.66 to 1.25 mg/dL
Calcium	9.5	8.4 to 10 mg/dL
Glucose	381	82 to 115 mg/dL
Total Bilirubin	5.6	0.1 to 1.2 mg/dL
ALP	284	56 to 119 U/L
AST	49	17 to 59 U/L
ALT	102	0 to 50 U/L
Sodium	131	136 to 145 mmol/L
Potassium	3.8	3.5 to 5.1 mmol/L
Cholride	95	99 to 112 mmol/L
Bicarbonate	33	21 to 33 mmol/L
Anion Gap	14	10 to 14 mmol/L

The chest X-ray was unremarkable. Given elevated liver enzymes, an RUQ ultrasound was done, which showed an irregularly shaped gallbladder with wall thickening (10 mm) filled with non-shadowing solid material, which is essentially avascular, and no acute cholecystic fluid and chronic dilation of the common bile duct measuring up to 8 mm. However, these findings were similar to imaging done four years ago. CT abdomen and pelvis without contrast showed layering sludge/debris within the gallbladder with a thickened gallbladder wall. 

**Figure 1:**
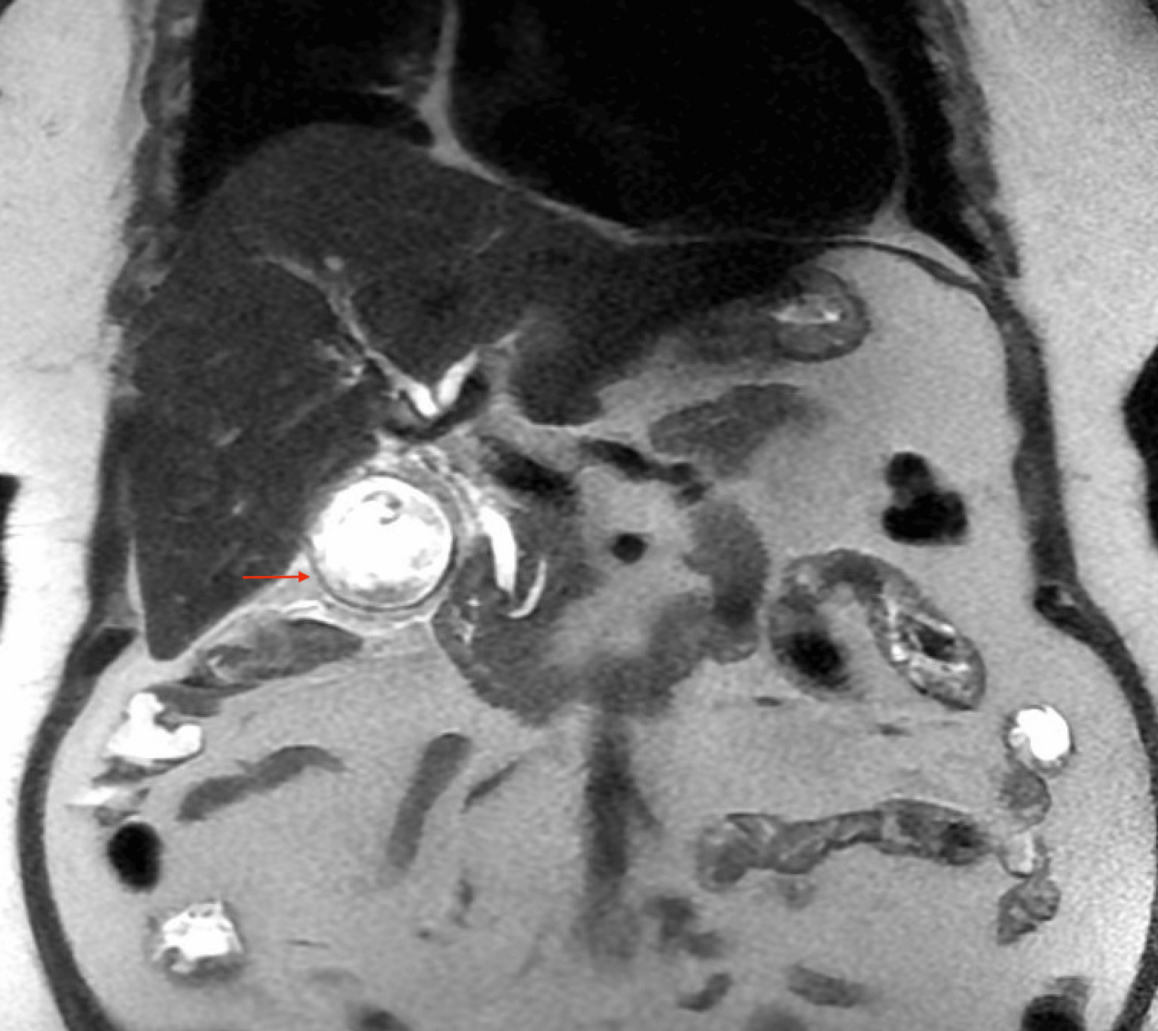
MRCP (coronal view) showing distended gallbladder with wall thickening suggestive of acute cholecystitis MRCP: Magnetic resonance cholangiopancreatography

**Figure 2:**
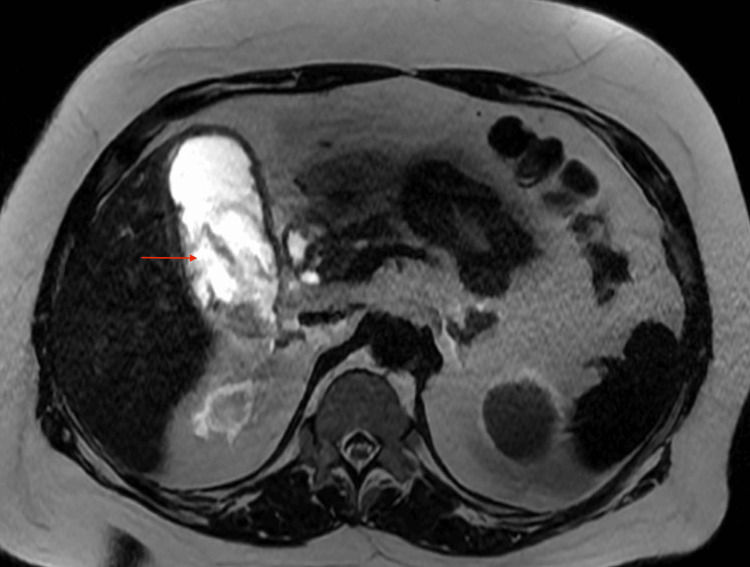
MRCP (axial view) showing distended gallbladder MRCP: Magnetic resonance cholangiopancreatography

Given elevated liver enzymes with a dilated common bile duct, magnetic resonance cholangiopancreatography (MRCP) was done to rule out choledocholithiasis. MRCP showed a distended gallbladder with wall thickening and edema containing cholelithiasis and sludge, mild pericholecystic fat stranding, and no intra- or extrahepatic biliary duct dilatation, with the common bile duct measuring up to 6 mm (Figures 1, 2).

These findings were suggestive of acute cholecystitis. Surgery was consulted for cholecystectomy. Given no abdominal pain and the patient's preference for conservative management, he was managed with antibiotics. His hiccups improved over the next three days, and he was discharged home with antibiotics to complete a seven-day course and advised to follow up outpatient with surgery.

Intractable hiccups can have a wide range of differentials, and leading to diagnosis in the absence of other symptoms can be challenging. Initial differentials for persistent hiccups include gastrointestinal, central nervous system, thoracic, metabolic, and neurological causes. In our case, some of the differentials, including gastroesophageal reflux, pancreatitis, hepatic pathology, central nervous system lesions (e.g., stroke or tumor), pulmonary embolism, myocardial infarction, pneumonia, and metabolic disturbances (e.g., electrolyte imbalances, renal failure), were systematically ruled out based on our patient's clinical presentation, stable vital signs, normal neurological examination, unremarkable chest X-ray, and absence of metabolic derangements in laboratory testing. Imaging (ultrasound and MRCP) revealed findings suggestive of acute cholecystitis. After the exclusion of all the possible differentials for hiccups and radiographic evidence available, acute cholecystitis was considered the most likely etiology of persistent hiccups in our patient. The resolution of hiccups following antibiotic therapy further supported the hypothesis that diaphragmatic irritation from gallbladder inflammation was the underlying cause, highlighting the importance of maintaining a broad differential diagnosis for atypical presentations such as isolated hiccups.

## Discussion

Acute cholecystitis is primarily caused by gallstones, which cause obstruction of the cystic duct and gallbladder inflammation [10]. While the classic presentation includes RUQ abdominal pain, tenderness, fever, and leukocytosis, variations in the clinical presentation have been well documented [5-11]. This is particularly relevant for elderly patients with comorbidities like diabetes mellitus, which can suppress the immune response and hence present with atypical symptoms like malaise, nausea/vomiting, confusion, or chest pain [5,8,11]. However, hiccups as the sole presenting symptom are exceedingly rare and sparsely reported in the medical literature [5].

As per the Tokyo guidelines, diagnosis of acute cholecystitis requires the presence of signs of local inflammation (Murphy’s sign or RUQ pain/mass/tenderness) and systemic inflammation (fever, elevated C-reactive protein [CRP], or elevated WBC count) confirmed by imaging findings of cholecystitis [12]. Moreover, Murphy's sign-on physical exam has a specificity as high as 97% [13]. Despite this, Trowbridge et al. [7] in their meta-analysis found that no single clinical or laboratory test had a significantly high positive or low negative likelihood ratio (LR) to rule in or rule out acute cholecystitis. Murphy's sign had a positive LR of 2.8, and RUQ tenderness had a negative LR of 0.4; however, the result was not statistically significant [7].

Hiccups, or singultus, result from sudden involuntary contraction of the diaphragm and intercostal muscle followed by closure of the glottis [14]. Transient hiccups are common and benign, but persistent hiccups indicate underlying pathology, like metabolic disturbances, central nervous system disorders, and gastrointestinal diseases [15]. The mechanism of hiccups includes a complex neurological reflex pathway consisting of peripheral and central midbrain pathways with sensory receptors located in the stomach, esophagus, and undersurface of the diaphragm, which can be stimulated by irritation/ distension [14,16]. The pathophysiology of hiccups in acute cholecystitis remains unclear. One proposed mechanism is diaphragmatic irritation from gallbladder inflammation, as seen in the case reported by Safa et al. [5], where gangrenous cholecystitis presented solely with intractable hiccups and no classic abdominal symptoms. Other possible mechanisms include vagus or phrenic nerve involvement or autonomic dysfunction.

Diagnostic confirmation is done with imaging, which shows signs of inflammation and thickening of the gallbladder wall [1]. In our case, MRCP, which was done to rule out choledocholithiasis, showed evidence of acute cholecystitis with gallbladder wall thickening and edema along with pericholecystic fat stranding. Management traditionally involves surgical cholecystectomy [1]. However, in select cases, including elderly patients with multiple comorbidities, mild disease, or surgical contraindications, conservative management with antibiotics can be used as an alternative approach. Our patient opted for non-surgical management and showed significant improvement with antibiotics alone, with subsiding inflammation contributing to the cessation of hiccups, further emphasizing the role of individualized treatment strategies.

## Conclusions

In conclusion, this case illustrates the variable clinical presentation of acute cholecystitis and the importance of maintaining a broad differential and using imaging modalities appropriately to facilitate timely diagnosis and appropriate management. This becomes even more important in elderly patients with comorbidities, which can suppress the immune response, leading to atypical clinical manifestations of different diseases.
